# Anti-Tumor Strategies of Photothermal Therapy Combined with Other Therapies Using Nanoplatforms

**DOI:** 10.3390/pharmaceutics17030306

**Published:** 2025-02-26

**Authors:** Rubing Xu, Shengmei Wang, Qiuyan Guo, Ruqian Zhong, Xi Chen, Xinhua Xia

**Affiliations:** 1 School of Pharmacy, Hunan University of Chinese Medicine, Changsha 410208, Chinagg1026kk@126.com (Q.G.);; 2 The First Hospital of Hunan University of Chinese Medicine, Changsha 410007, China; 3 Hunan Provincial Center for Drug Evaluation and Adverse Reaction Monitoring, Changsha 410013, China; 19976689333@163.com

**Keywords:** cancer therapy, photothermal therapy, synergistic therapy, pre-clinical and clinical studies

## Abstract

Conventional cancer treatments often have complications and serious side effects, with limited improvements in 5-year survival and quality of life. Photothermal therapy (PTT) employs materials that convert light to heat when exposed to near-infrared light to raise the temperature of the tumor site to directly ablate tumor cells, induce immunogenic cell death, and improve the tumor microenvironment. This therapy has several benefits, including minimal invasiveness, high efficacy, reduced side effects, and robust targeting capabilities. Beyond just photothermal conversion materials, nanoplatforms significantly contribute to PTT by supplying effective photothermal conversion materials and bolstering tumor targeting to amplify anti-tumor effects. However, the anti-tumor effects of PTT alone are ultimately limited and often need to be combined with other therapies. This narrative review describes the recent progress of PTT combined with chemotherapy, radiotherapy, photodynamic therapy, immunotherapy, gene therapy, gas therapy, chemodynamic therapy, photoacoustic imaging, starvation therapy, and multimodal therapy. Studies have shown that combining PTT with other treatments can improve efficacy, reduce side effects, and overcome drug resistance. Despite the encouraging results, challenges such as optimizing treatment protocols, addressing tumor heterogeneity, and overcoming biological barriers remain. This paper highlights the potential for personalized, multimodal approaches to improve cancer treatment outcomes.

## 1. Introduction

Cancer remains one of the top causes of death globally, with approximately 19.3 million new cases and 10 million fatalities reported in 2020 [1]. Despite advancements in early cancer detection and treatment, the variability and adaptability of tumors pose significant challenges in achieving effective outcomes. Many cancers are diagnosed at intermediate or advanced stages, with metastasis complicating complete tumor elimination through surgery. Treatments like chemotherapy and radiotherapy (RT) have improved patient outcomes but can lead to invasive risks, systemic toxicity, and treatment resistance due to the rapid replication and mutation of tumors [2]. The development of new drugs, particularly for tumor types resistant to conventional treatments, encounters several challenges, including the intricate design of linker structures, difficulties in drug delivery, and the risk of off-target effects. Given the complexity of cancer, there is an urgent need for more precise, effective, and less harmful therapeutic approaches.

Photothermal therapy (PTT), as a cancer treatment method, has broad application prospects because photothermal agents (PTAs) can selectively accumulate in tumor tissues to generate heat at specific wavelengths of light [3]. PTT is expected to be a promising approach for the treatment of cancer due to its advantages of minimal invasiveness, high spatial and temporal selectivity, and low systemic side effects compared with conventional therapies [4]. Specifically, this localized heating can cause direct tumor cell death, alter the tumor microenvironment (TME), and trigger various biological responses that contribute to the overall therapeutic effect [5]. For instance, near-infrared (NIR) light (usually within the 650–900 nm range) is preferred for its ability to penetrate deeper tissues and its minimal absorption by water and hemoglobin (Hb), making it suitable for targeting deeper tumors [6]. PTT includes traditional PTT (≥45 °C) and mild PTT. Traditional PTT is effective for tumor ablation but can harm healthy tissues due to imprecise PTA targeting and laser risks. Mild PTT minimizes damage to normal tissues and immune responses but is less effective against tumors [7]. Therefore, the combination of PTT with other treatment modalities, such as chemotherapy, RT, photodynamic therapy (PDT), immunotherapy, and gene therapy, has been developed and can leverage synergistic effects, overcome treatment resistance, and address the heterogeneity of tumors [8]. The development of advanced nanomaterials has played a crucial role in enabling these combination therapies. Nanoparticles (NPs) can serve as multifunctional platforms, simultaneously acting as PTAs, drug carriers, and even diagnostic tools [9]. This versatility allows for the formulation of sophisticated treatment strategies that can target multiple aspects of cancer biology simultaneously.

**Figure 1:**
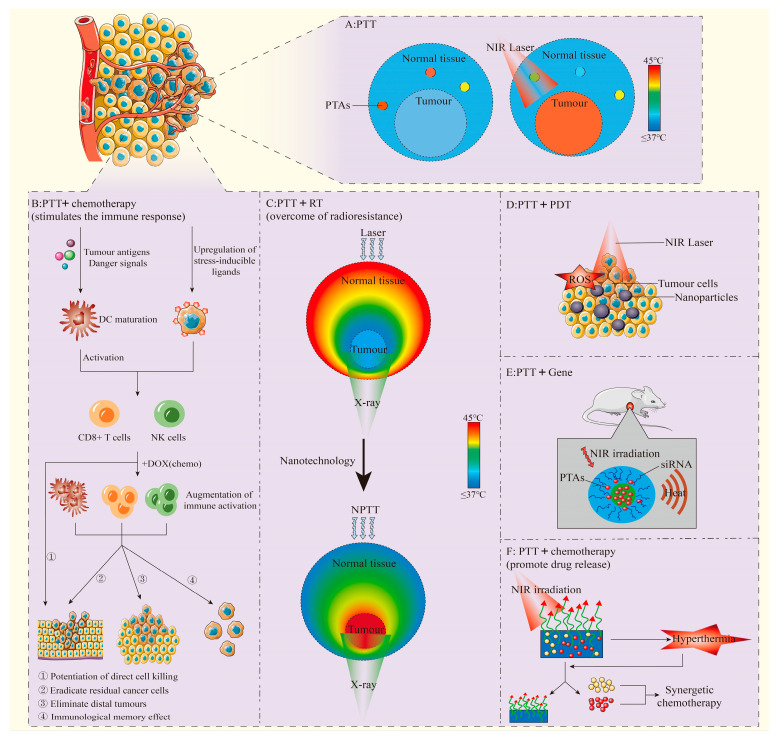
(**A**): PTT performs photothermal conversion through PTAs to increase local temperature. Adapted from [10], Institute of Electrical and Electronics Engineers, 2023. (**B**): PTT combined with chemotherapy stimulates anti-tumor immune response in vivo, and can play a synergistic anti-tumor effect. Adapted from [11], Springer Nature, 2018. (**C**): Effects of different types of PTT. Adapted with permission from [12], Elsevier, 2016. (**D**): PTT and PDT combined anti-tumor. Adapted from [13], Multidisciplinary Digital Publishing Institute, 2018. (**E**): PTT combined with Gene therapy. Adapted with permission from [14], American Chemical Society, 2011. (**F**): PTT promotes specific release of chemotherapy drugs and exerts synergistic anti-tumor effects. Adapted from [15], Springer Nature, 2023.

This narrative review examines the integration of PTT with other therapeutic approaches (e.g., chemotherapy, RT), leveraging the multifunctional capabilities of nanoplatforms to boost tumor treatment effectiveness (Figure 1). The goal is to address PTT’s limitations through combination therapies, enhancing treatment outcomes, minimizing side effects, and promoting the advancement of nanomedicine to offer fresh insights for clinical applications.

## 2. Photothermal Therapy

### 2.1. Operating Principles

**Figure 2:**
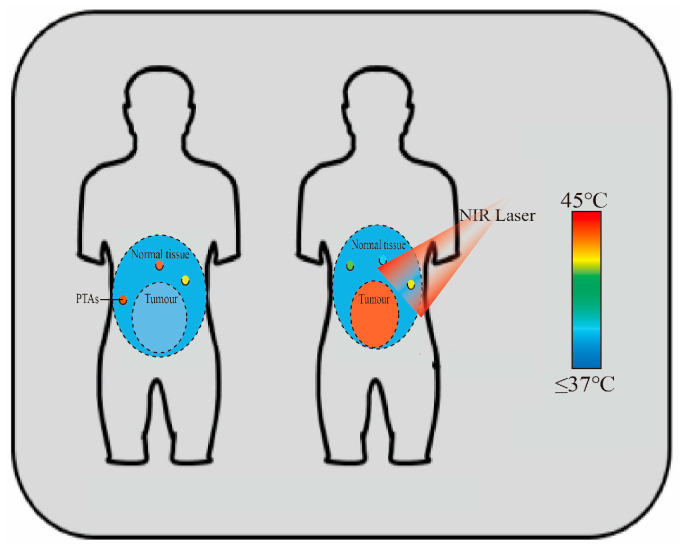
PTT performs photothermal conversion via PTAs, which converts light energy into heat, raising the TME temperature to 41–47 °C or higher. Adapted from [10], Institute of Electrical and Electronics Engineers, 2023.

PTT uses light-to-heat conversion via PTAs. When PTAs are exposed to light, usually in the NIR region (650–900 nm), they absorb photon energy and convert it into heat through non-radiative relaxation [16]. This localized heating can elevate the temperature of the TME to 41–47 °C or higher (Figure 2), inducing hyperthermia and subsequent cell death [10]. When tissue temperature reaches 41 °C, cell diffusion increases and blood flow accelerates, triggering heat-shock protein production to repair minor heat damage. At 42 °C, tissue damage becomes irreversible. Sustained temperatures of 42–46 °C for 10 min cause cell necrosis, while 46–52 °C induces microvascular thrombosis, leading to rapid tissue ischemia and cell death. Above 60 °C, cells die almost instantly. In clinical settings, PTAs are typically administered intravenously to induce therapeutic effects, entailing the following steps: (1) PTAs are injected intravenously into patients; (2) PTAs circulate through the body; (3) they selectively accumulate in tumors via active/passive targeting or molecular activation (e.g., protease activity, anoxic conditions); (4) tumor exposure to specific-wavelength light excites PTAs; (5) this excitation causes thermal damage and tumor ablation [17].

### 2.2. Influence Factors

#### 2.2.1. Light Wavelength

The choice of light wavelength is crucial for PPT, and NIR light is generally selected because it is able to penetrate deeper tissues and achieve treatment of tumors at deeper sites. NIR light within the 650–1700 nm range has lower energy and has deeper tissue penetration ability than visible light, because of its longer wavelength and less scattering and reabsorption. The effective depth to which NIR–I (650–950 nm) light can penetration into tissues is less than 1 cm, while NIR-II (1000–1700 nm) light can penetrate 3–5 cm, suggesting that tumors in deep tissue can be treated more effectively with a light source in the NIR-II region. Weissleder and Ntziachristos demonstrated that NIR light could penetrate several centimeters into biological tissues, making it suitable for non-invasive imaging and therapy [18]. Additionally, NIR light falls within the “biological window”, where the absorption by endogenous chromophores like water and Hb is minimal, reducing the damage to healthy tissues and resulting in the lower background fluorescence from biological tissues [19]. NIR light can be generated by LED arrays or lasers. Lasers provide high-efficiency, coherent light with strong tissue penetration and focused energy transfer. When selecting an NIR light source, safety and biocompatibility are important. For example, the maximum permissible exposure (MPE) for an 808 nm laser is 0.33 W/cm^2^, while for 980 nm and 1064 nm lasers, the MPE is 0.72 W/cm^2^ and 1.0 W/cm^2^, respectively.

#### 2.2.2. Photothermal Agent

PTAs are substances capable of absorbing a specific wavelength of light (usually NIR) and converting it into heat energy. PTAs are key to PTT due to their high photothermal conversion efficiency, biocompatibility, and easy functionalization with drugs and photosensitizers. Various PTA materials have been explored, classified by chemical structure into metallic nanomaterials, carbon-based nanomaterials, organic dyes, and conjugated polymers. They can also be categorized as natural or synthetic photosensitizers, and by excitation mode as single or multiple excitation photosensitizers [20,21,22,23,24,25,26]. (Table 1) Noble metal nanoparticles, including of gold nanorods, nanoshells, and nanocages can exhibit tunable optical properties and high photothermal conversion efficiency [27]. Huang et al. illustrated that gold nanorods could achieve photothermal conversion efficiencies of up to 95%, making them highly effective for PTT [28]. Carbon-based substances–for instance, carbon nanotubes and graphene oxide (GO)—offer large surface areas and high thermal conductivity [29]. Yang et al. showed that PEGylated GO nanoparticles were able to bring about tumor ablation in mice with minimal adverse effects [30]. Organic compounds, like indocyanine green (ICG), with strong photodynamic properties and certain photothermal properties as well as porphyrin derivatives have good biocompatibility and potential with regard to photothermal and photodynamic effects [31]. Prussian blue nanoparticles (PBNPs) have high photothermal conversion efficiency and are widely used in tumor treatment. Cano-Mejia, J. et al. reported that PBNPs, when irradiated by an 808-nm laser, rapidly heated to above 40 °C, reducing tumor growth in mice [32]. Sur, S. et al. developed PEG-modified PBNPs (60 nm) for tumor treatment in mice, showing significant tumor reduction with no toxicity over two months [33]. Xue et al. created a composite nano-drug platform by loading ICG on magnetic PBNPs, improving circulation and targeting tumor tissues via the EPR effect and magnetic targeting. In a nude mouse model, the light-induced photothermal/photodynamic effect effectively inhibited tumor growth, indicating strong clinical potential [34]. Transition metal compounds, such as copper sulfide and molybdenum disulfide, showed strong NIR absorption and were capable of being easily functionalized for multimodal applications [35].

#### 2.2.3. Nanoplatforms

##### Common Nanoplatforms

Nanoplatforms, including various NPs, are key tools for PTT, enhancing PTA delivery to tumor tissues and improving PTT selectivity and effectiveness. Common types include polymeric, liposomal, micellar, inorganic, virus-like, and extracellular vesicle nanoparticle platforms. Their classifications and characteristics are summarized in Table 2 [36,37,38,39,40,41]. The targeting efficiency of nanoplatforms can be improved through various approaches. For passive targeting, the EPR effect can be utilized to encourage the preferential accumulation of NPs or macromolecules in tumor tissues by adjusting their size and surface characteristics. Active targeting, in contrast, often relies on high-affinity ligands that bind to specific surface molecules predominantly found on cancer or tumor epithelial cells. Several active targeting ligands for PTT drugs have been identified, including peptides, proteins, and nucleic acid aptamers [17].

##### Self-Assembled Nanoplatforms

In recent years, self-assembled nanoplatforms have attracted extensive attention because of their high structural controllability, stability, and versatility. Self-assembly refers to the spontaneous formation of ordered junctions by molecules or NPs through weak interactions (such as van der Waals forces, hydrogen bonds, electrostatic interactions, etc.). In the application of PTT, the self-assembled nanoplatform can achieve targeted drug delivery through the modification of specific ligands, enhance the aggregation at the tumor site, and use metal or carbon-based materials (such as gold NPs and graphene) to enhance the photothermal effect and enhance the anti-tumor effect [41]. In addition, studies have shown that self-assembled nanoplatforms can also be used in combination with chemotherapy, RT, and other therapeutic means to produce synergistic effects [42].

### 2.3. Anti-Tumor Mechanism

#### 2.3.1. Inducing Tumor Cell Death

The mechanisms by which PTT leads to tumor cell death are diverse and depend on the temperature achieved and the duration of hyperthermia. Rapid and substantial temperature elevation (>50 °C) results in immediate disruption of the cell membrane, protein denaturation, and cellular disintegration through necrosis [43]. Moderate hyperthermia (41–45 °C) can trigger programmed cell death pathways, including apoptosis, the death receptor pathway (which is the external pathway), the mitochondrial dependence pathway (internal pathway), the endoplasmic reticulum-related death pathway, and autophagy. Roti et al. provided a comprehensive review of cellular responses to hyperthermia, highlighting the complex interplay between various cell death mechanisms [44].

#### 2.3.2. Promoting Anti-Tumor Immune Response

PTT induces the release of damage-associated molecular patterns (DAMPs), triggering anti-tumor immune responses via immunogenic cell death. It activates a systemic immune response, including immune cell redistribution, cytokine release, and memory T lymphocyte activation. Kroemer et al. highlight thermal stress leading to calreticulin exposure, adenosine triphosphate (ATP) release, and HMGB1 secretion, which activate dendritic cells and stimulate T cell responses [45]. Transcriptome analysis revealed changes in gene expression and immune cell composition in B16-F10 tumors following PTT treatment. A total of 256 differentially expressed genes were identified, with 215 being downregulated and 41 being upregulated. Functional annotation indicated that most of these genes were involved in immune and inflammatory responses. Immune cell composition analysis also showed significant alterations post-PTT, including changes in regulatory T cells, M2 macrophages, and B cells.

#### 2.3.3. Other Anti-Tumor Mechanisms

In addition to the above mechanisms, the thermal effect of PTT can damage the blood vessels surrounding the tumor, leading to vascular leakage and blood flow disruption, reducing the nutrient supply of tumor cells so as to achieve anti-tumor effects [46]. Secondly, when PTT is combined with a heat-sensitive drug delivery system, local high temperatures can promote drug release and enhance local therapeutic effects, which is especially suitable for combination chemotherapy and immunotherapy [47]. In addition, when PTT is combined with PDT, the photothermal effect can also enhance the production of reactive oxygen species (ROS) and further promote tumor cell death [48].

Together, understanding these mechanisms is crucial for optimizing PTT protocols and designing effective combination strategies. That PTT can induce multiple cell death pathways and stimulate responses of immune provides a strong rationale for its use in combination with other treatment modalities to enhance the comprehensive therapeutic effect.

### 2.4. Development and Application

**Figure 3:**
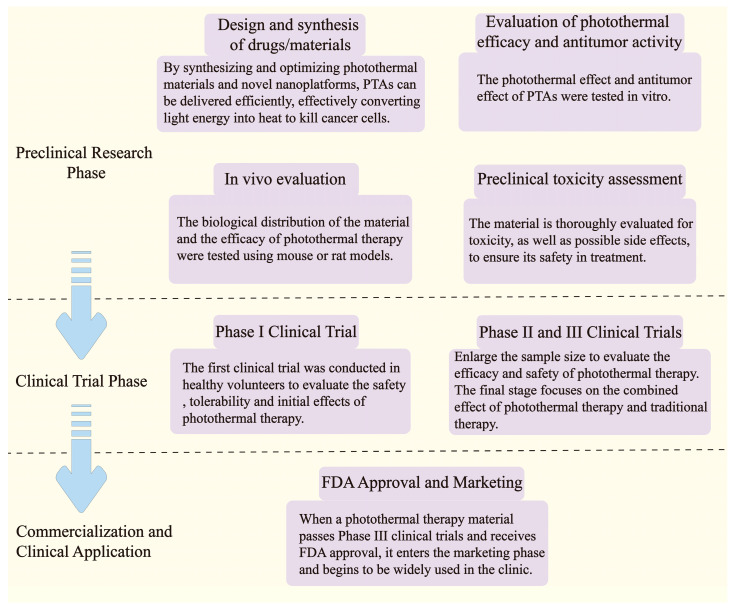
Flowchart of PTT development and application and possible obstacles.

The development and application of PTT require several stages, including preclinical research, animal model testing, clinical trials, and clinical application, each with its own challenges (Figure 3). Issues such as the biocompatibility and toxicity of materials, photothermal efficiency and selectivity, drug delivery efficiency, and side effect management must be addressed one by one to progress to clinical application. For example, Zhu, H., et al. significantly improved biocompatibility through the surface modification of gold NPs [49]; Li, Y., et al. developed polymer nanoparticles that can target tumors and enhance photothermal effects, improving the selectivity and therapeutic effects of PTT [50]; in clinical trials, Chen, Q., et al. effectively improved treatment outcomes by combining immunotherapy [51]. Overall, by optimizing material design, enhancing biocompatibility, improving targeting, and integrating with other treatment modalities, it is possible to overcome bottlenecks throughout the process and facilitate the transition of PTT to clinical application and market promotion.

## 3. Photothermal Therapy in Combination with Other Therapeutic Modalities

It is hard to completely eliminate solid tumors by using PTT alone. Consequently, it is imperative to combine PTT with other treatment methods to leverage the advantages of each treatment approach, thereby generating additional or even synergistic therapeutic effects. Nanotechnology can offer a technical platform for the combination of multiple methods and plays an important role in combination therapy: (1) Nanotechnology can improve the basic properties and biological activities of drug molecules, increase the bioavailability of drugs with poor water solubility and enhance the selective accumulation of drugs in tumors; (2) Because of its unique size advantage and performance, nanocarriers can effectively enhance the targeted delivery effectiveness of therapeutic drugs and minimize the off-target rate; (3) Nanomaterials can achieve targeted drug delivery through functionalization, and use the characteristics of the TME (such as pH change and enzyme activity) as trigger factors to achieve controlled drug release in tumor sites; (4) Nanotechnology can combine diagnostic and therapeutic functions into a kind of NP and form a therapeutic and diagnostic nanomedicine platform, which can monitor pharmacokinetics, drug accumulation, and disease progression, further advancing the study of tumor heterogeneity and patient-specific characteristics, ultimately contributing to the development of personalized medicine; (5) Nanoplatforms can transport several active drugs at the same time, promote synergistic therapy, and avoid some drug resistance mechanisms. With the continuous development of PTT and nanotechnology, the combination of PTT with other therapies (including chemotherapy, RT, immunotherapy, gene therapy, etc.) has been extensively explored and shown good application prospects for tumor treatment. Table 3 summarizes the anti-tumor mechanisms and characteristics of PTT combined with different therapeutic methods.

### 3.1. Combination of PTT with Chemotherapy

#### 3.1.1. Promotion of Drug Uptake and Accumulation

Hyperthermia induced by PTT can enhance cellular membrane permeability, promoting drug internalization and increasing drug uptake. Liang et al. showed that mild hyperthermia was capable of increasing the cellular uptake of by up to 2.5-fold in breast cancer cells [52]. Local heating can improve blood flow and vascular permeability in the tumor, facilitating the distribution and accumulation of drugs in the tumor site. Li et al. showed that PTT-induced mild hyperthermia could increase tumor perfusion by 1.4-fold and enhance the accumulation of liposomal doxorubicin (DOX) in tumors by 2.6-fold [53]. Zhang et al. introduced a multifunctional optical fiber-based drug delivery and controlled release system designed to improve tumor vascular permeability and modulate the TME, facilitating better drug penetration throughout the tumor. Additionally, the drug-loaded fused agarose gel particles could remain attached to the tumor for an extended period, continuously delivering DOX and achieving an enhanced drug release rate to further target and destroy tumor cells [54].

#### 3.1.2. Synergy of Anti-Tumor Effects

**Figure 4:**
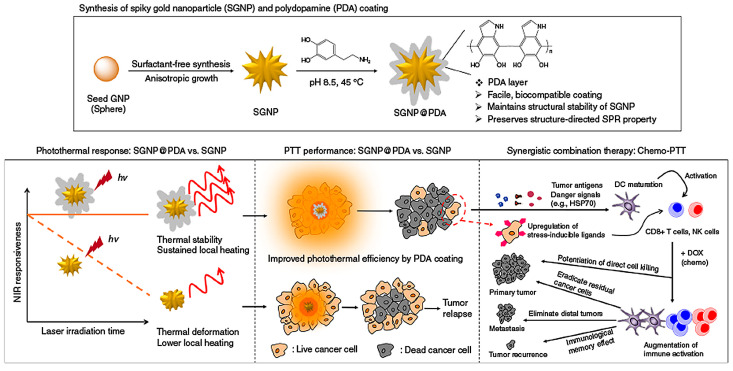
The combination of PTT and chemotherapy triggers a powerful anti-tumor immune response in vivo, producing a synergistic anti-tumor effect. Reproduced from [11], Springer Nature, 2018.

PTT combined with chemotherapy can exert a synergistic anti-tumor effect. Yao et al. designed graphene quantum dot-capped mesoporous silica NPs for combining PTT with controlled drug delivery, achieving significant tumor growth inhibition in vivo [55]. Xue et al. developed PBNPs encapsulated with a gel-adriamycin conjugate, where the drug release was activated by gelatinase, an endogenous proteolytic enzyme that is highly expressed in tumor tissues. In vitro experiments on Huh7 tumor cells demonstrated that the material exhibited strong photothermal effects and showed a synergistic therapeutic effect when combining PTT and chemotherapy [34]. Liu et al. prepared a nano-MoS_2_ by the hydrothermal method, which had good photothermal conversion performance, a high DOX loading rate, and pH-sensitive drug-controlled release ability. The inhibition rate of MoS_2_-DOX nanocomposites loaded with DOX on HuH-7 cells was as high as 92.09% under near-infrared irradiation, which was considerably more effective than PTT or chemotherapy individually [56]. Gong et al. grafted thyrocalcitonin (TCA) and folic acid (FA) onto Fe_3_O_4_-modified graphene oxide (MGO) to prepare a new hybrid nanomaterial MGO-TCA-FA and successfully loaded DOX onto MgO-TCA-FA. In vitro and in vivo experiments showed that the nano-drug carrier could specifically target liver cancer cells and had a significant killing effect, and that the tumor inhibition rate of PTT combined with chemotherapy was about 85%, which was significantly higher than that of single therapy [57]. Nam et al. developed gold nanorods conjugated with CpG oligonucleotides, demonstrating enhanced dendritic cell activation and anti-tumor immunity when combined with PTT (Figure 4) [11]. Chen et al. designed a nanoplatform by coating mesoporous polydopamine (mPDA) on gold nanorods and loading DOX into the Au@mPDA system. This platform exhibited a high drug loading capacity, efficient photothermal conversion, and a dual-responsive drug release triggered by both pH and photothermal effects. In vivo studies revealed that the Au@mPDA@DOX system notably improved anti-tumor outcomes in breast cancer compared to chemotherapy or PTT alone [58].

#### 3.1.3. Enhancement of the Responsiveness of Tumor Cells to Chemotherapy Drugs

Enhancing the sensitivity of tumor cells to chemotherapy is crucial for improving therapeutic outcomes, minimizing drug resistance, reducing side effects, enabling personalized treatment, and developing novel combination therapies. Some chemotherapy drugs exhibit minimal cytotoxicity at 37 °C, but their chemical structure can undergo changes when exposed to heat, leading to increased toxicity to cells. Mild hyperthermia can enhance the effectiveness of chemotherapy by inhibiting DNA repair mechanisms, altering cellular metabolism, and regulating the expression of heat shock proteins, thereby sensitizing cancer cells to the drugs [59]. When cyclophosphamide, cisplatin, carboplatin, carbamazepine, and other chemotherapeutic drugs act synergistically with phototherapy on cancer cells, the cytotoxicity of these drugs shows a linear increase as the local tumor temperature rises from 37 °C to 40 °C, and the toxicity of chemotherapeutic drugs can be enhanced to the greatest extent when the local temperature rises to 40.5~43.0 °C [60]. Houdaihed et al. studied a kind of polymer nanoparticle for the co-delivery of paclitaxel and everolimus, and their half-lives in vivo were 5.8 h and 30 h, respectively; the optimal ratio of paclitaxel and everolimus in vivo could be maintained at 1:0.5 by preparing polymer nanocarriers so as to accurately control the ratio of combined drugs in tumor sites and the combined anti-tumor effect [61]. Ni et al. explored the mechanism by which hyperthermic intraperitoneal chemotherapy improves cellular sensitivity to chemotherapeutic drugs. They examined the effects of hyperthermic chemotherapy on the heat-sensitive ovarian cancer cell line A2780 using quantitative proteomics and validated the findings with Western blot analysis. Both the quantitative proteomics results and Western blot analysis revealed that hyperthermic chemotherapy upregulates the expression of retinoblastoma-like protein 1 (RBL1/p107). The study confirmed that PTT influences DNA repair mechanisms by modulating the synthesis of proteins involved in DNA damage repair, thus enhancing cellular sensitivity to chemotherapeutic agents [62].

#### 3.1.4. Overcome of Multidrug Resistance

Multi-drug resistance is one of the important reasons for the failure of chemotherapy, and it could be overcome by high temperature leading to mitochondrial dysfunction, inhibiting the production of ATP and thereby reducing the efflux of therapeutic drugs in tumor cells. Issels reviewed the molecular mechanisms underlying the chemosensitizing effects of hyperthermia, highlighting its potential to overcome drug resistance [63]. Furthermore, PTT can help overcome multidrug resistance by modulating the expression and function of drug efflux pumps and altering the TME. Xu et al. demonstrated that gold nanorod-mediated PTT could significantly reduce the expression of P-glycoprotein in drug-resistant breast cancer cells, enhancing the efficacy of DOX [64]. Tu et al. synthesized nano-graphite flakes loaded with the chemotherapy drug DOX in a pH-sensitive manner, modifying the nanoparticles with hyperbranched polyglycerol amine. They also attached a mitochondrial targeting ligand, triphenylphosphine, to the surface. When exposed to NIR laser irradiation, the high heat energy generated by the system disturbed mitochondrial function, inhibited ATP production, and reversed multi-drug resistance in cancer cells [65]. In a successfully synthesized mPDA that could efficiently load DOX and drug efflux inhibitor TPGS (D-α-tocopherol polyethylene glycol 1000 succinate), NIR irradiation promoted nanoparticle escape from the endosome, facilitating the release of drugs into the cytoplasm, thereby overcoming tumor multi-drug resistance and enhancing cytotoxicity against MCF-7/ADR cells [66].

#### 3.1.5. Pre-Clinical Studies

**Figure 5:**
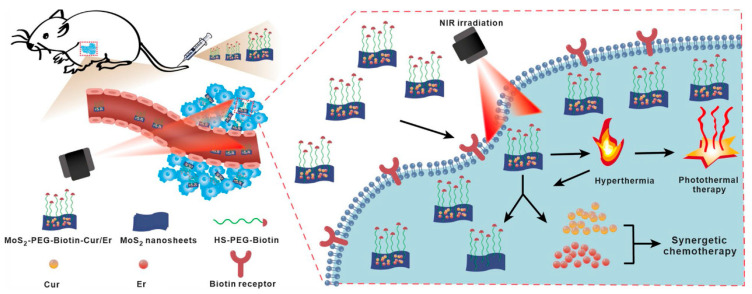
A schematic of MoS_2_-PEG-Biotin as a carrier demonstrates its capability for the targeted delivery of curcumin (Cur) and erlotinib (Er), facilitating the combination of synergistic chemotherapy and PTT. Reproduced from [15], Springer Nature, 2023.

Several preclinical studies have highlighted the enhanced efficacy of PTT–chemotherapy combinations. Chen et al. [15] developed a targeted co-delivery system using MoS_2_ nanosheets to deliver curcumin and erlotinib for synergistic lung cancer treatment (Figure 5). This innovative combination approach shows promise for clinical translation. Yang et al. reported complete tumor eradication and 100% survival over 40 days using a GO-based nanoplatform loaded with DOX in a mouse model of colorectal cancer [67]. Nowadays, clinical translation of these combination approaches is ongoing. Additionally, clinical trials are underway to evaluate the combination of AuroLase^®^ therapy (gold nanoshells) with standard chemotherapy for advanced solid tumors [68]. Preliminary results have shown promising safety profiles and potential efficacy, paving the way for further clinical investigations.

### 3.2. Combination of PTT with Immunotherapy

PTT induces tumor cell death, releasing cell fragments and tumor-associated antigens that trigger immune responses [69]. However, immune stimulation from PTT alone is often insufficient. Combining PTT with immune adjuvants or immunosuppressants can enhance immune cell infiltration and maturation in tumors, boosting immune-related cytokine production in peripheral blood. Immunosuppressants can counteract negative regulatory signals, thereby enhancing the effectiveness of PTT. The integration of PTT with immunotherapy has become a potent strategy for boosting anti-tumor immune responses and achieving durable tumor control. This approach utilizes PTT-induced immunogenic cell death, which triggers the release of DAMPs like calreticulin, ATP, and HMGB1. These DAMPs activate dendritic cells, facilitate T cell priming, and strengthen the immune response, ultimately improving the overall anti-tumor efficacy [46]. This immune-stimulating effect of PTT provides a strong rationale for its combination with various immunotherapeutic strategies. Several immunotherapy approaches have been explored in combination with PTT, including immune adjuvants and immune checkpoint inhibitors.

#### 3.2.1. PTT in Combination with Immune Adjuvants

Wang et al. developed a nanoparticle-based system that integrated PTT with a toll-like receptor agonist, successfully inducing anti-tumor immunity in a breast cancer model [70]. The combination therapy not only eradicated primary tumors but also reduced the growth of distant, untreated tumors by 67%, demonstrating its potential for systemic anti-tumor effects. On the other hand, Chen et al. designed a nanoparticle delivery system that encapsulated the immune stimulant resiquimod (R848@NPs) within a core made of hydrophobic polyaniline and hydrophilic glycol-chitosan. Upon exposure to NIR laser irradiation, R848@NPs facilitated the infiltration of CD3+ T cells and the secretion of granzyme B, as well as increased the pro-inflammatory cytokine IL-6 and reduced the immunosuppressive IL-10 within the TME. These results indicate that the combined therapy not only promotes tumor cell apoptosis but also mitigates immunosuppression, helping to prevent tumor recurrence and metastasis [71]. Shen et al. designed G5-PBA@CuS/cGAMP nanoparticles, which possess the following advantages: (1) The constructed NPs had outstanding photothermal conversion efficiency and protein adsorption characteristics, and could be used for photothermal/immunotherapy of tumors after being compounded with the STING activator cGAMP, thus inducing immunogenic death and activating the immune system; (2) After laser irradiation, the G5-PBA@CuS/cGAMP complex effectively integrates PTT and immunotherapy to suppress the primary tumor. Meanwhile, the modified phenylboronic acid (PBA) molecule captures primary tumor antigens, forms an in-situ vaccine, and triggers an adaptive immune response, which significantly hinders the growth of distant tumors. (3) The G5-PBA@CuS/antigen/cGAMP complex, prepared in vitro, can serve as a pre-formed nano-vaccine for immunotherapy of primary tumors. It also helps prevent tumor recurrence by generating immune memory [72].

#### 3.2.2. PTT in Combination with Immune Checkpoint Inhibitors

Chao et al. developed a nanoplatform that integrates gold nanoshells with anti-PD-L1 antibodies, showing greater tumor targeting and enhanced anti-tumor effects compared to conventional antibody therapy. This strategy led to a 3.2-fold improvement in tumor growth suppression and notably extended overall survival in a murine colon cancer model [73]. Cheng et al. used ansamitocin P3 (AP3), gold nanoclusters (AuNCs), and anti-programmed cell death-ligand1 (anti-PD-L1) to research and develop the multifunctional gold nanocapsule AP3-AuNCs-anti-PD-L1 for the treatment of hepatocellular carcinoma. This nanoplatform generated tumor-associated antigens and facilitated controlled AP3 release, promoting dendritic cell maturation, T cell activation, targeted tumor cell destruction, and the production of immunogenic tumor microenvironments, thereby enhancing immune responses [74]. The photothermal properties of black phosphorus (BP) can directly damage or eliminate tumor cells, promote the recruitment of monocytes to the tumor site, initiate an innate immune response, release tumor-specific antigens from necrotic cells, and activate a CTL-mediated adaptive immune response. Research has demonstrated that combining BP with a CD47 antibody significantly suppresses cancer cell proliferation, yielding a synergistic anti-tumor effect. This combination polarizes tumor-associated macrophages to the M1 phenotype and blocks the “don’t eat me” signal (CD47-SIRPα), enhancing macrophage-mediated phagocytosis. Activated macrophages also improve the cross-presentation of local tumor antigens, triggering the production of tumor-specific T cells that can target distant tumors with the same antigens, thereby offering the potential to combat metastatic cancer [75]. A separate study discovered that combining PTT with immune adjuvant nanoparticles, like anti-CTLA-4 therapy, boosts CD8+ CTL infiltration in secondary tumors, thereby enhancing the anti-tumor immune response. Furthermore, the combination of PTT with an immune checkpoint blockade (e.g., PD-L1 and CTLA-4) may provide superior effectiveness compared to blocking both simultaneously. However, simultaneous blockade could lead to immune-related adverse events or cytokine release syndrome, which may be intolerable for mice following PTT [5]. The clinical translation of these approaches is still in early stages, with ongoing trials assessing PTT with gold NPs in combination with immune checkpoint inhibitors for patients with advanced solid tumors [68]. Preliminary results have shown promising safety profiles and potential efficacy, with several patients exhibiting partial responses or stable disease.

### 3.3. Combination of PTT with Radiotherapy

RT is the main nursing standard for many locally advanced cancers, but cancer cells may develop radiation resistance. Hyperthermia, by raising the local tumor temperature through PTT, has been proven to be a powerful enhancer of RT. The main advantages of combining these two treatment schemes are that PTT drives to induce hyperthermia in tumor, at the same time, it increases blood flow in tumor tissue and improves oxygenation, thus making cells more sensitive to radiation. This combination strategy is based on several synergistic mechanisms, including radiosensitization, improved tumor oxygenation, and complementary cell killing mechanisms.

#### 3.3.1. Increase of Radiosensitivity

Horsman and Overgaard provided a comprehensive review of the potential of hyperthermia as a radiosensitizer, demonstrating that mild hyperthermia (41–43 °C) can notably boost the radiosensitivity of tumor cells, with a thermal enhancement ratio of 1.5:5 depending on the heating temperature and duration [76]. This synergistic effect is attributed to several mechanisms, including the inhibition of DNA repair, cell cycle synchronization, and increased oxidative stress. The hyperthermia induced via PTT can heighten the sensitivity of tumor cells to radiation by inhibiting DNA repair mechanisms and increasing oxidative stress. Krawczyk et al. demonstrated that mild hyperthermia (41–42.5 °C) inhibited BRCA2-dependent homologous recombination, leading to increased sensitivity to ionizing radiation [77], and observed a significant reduction in BRCA2 protein levels and impaired recruitment of RAD51 to DNA damage sites following hyperthermia treatment.

#### 3.3.2. Overcome of Radioresistance

**Figure 6:**
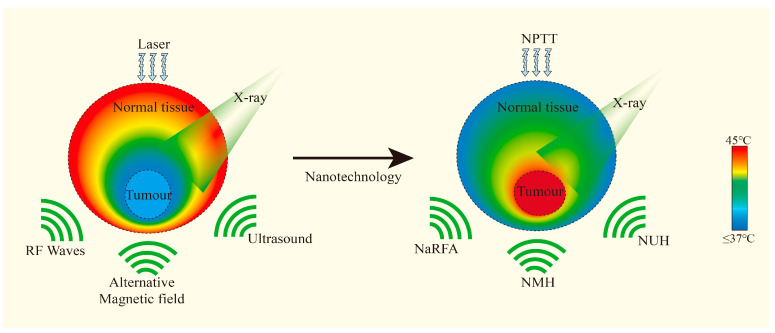
Different types of nanoparticles accumulate in the tumor and absorb various external energies, enhancing the effect of hyperthermia. Adapted with permission from [12], Elsevier, 2016.

Considering that RT primarily induces DNA damage, while PTT causes protein denaturation and membrane disruption, their complementary action can lead to more comprehensive tumor cell death [12], help overcome resistance mechanisms, and improve overall therapeutic efficacy. Song et al. showed that PTT-induced mild hyperthermia increased tumor oxygenation by 2.5-fold, leading to enhanced radiosensitivity in a mouse model of breast cancer [78]. This improvement in tumor oxygenation is particularly important for overcoming the radioresistance of hypoxic tumor regions, which are a major challenge in conventional RT. Beik et al. provided an extensive review of the nanotechnology-based approaches for combining hyperthermia with RT, highlighting the potential of multifunctional nanoplatforms to enhance treatment outcomes (Figure 6) [12].

#### 3.3.3. Development of Some Advanced Nanoplatforms

Advanced nanoplatforms have been developed to serve as dual-functional agents for both PTT and radiosensitization, including gold NPs, bismuth-based NPs, gadolinium-based NPs, and carbon-based nanomaterials. Hainfeld et al. showed that combining gold NPs with thermal therapy and RT resulted in a fourfold increase in long-term survival in a mouse model of squamous cell carcinoma compared to RT alone [79]. This enhanced effect was attributed to gold’s high atomic number, which improved X-ray photoelectric absorption, and the ability of gold NPs to generate heat under NIR irradiation. Song et al. developed PEGylated Bi_2_Se_3_ nanoplates that achieved a 3.2-fold increase in tumor growth inhibition when combined with RT compared to RT alone [80]. The high atomic number of bismuth contributed to effective radiosensitization, while the strong NIR absorption of Bi_2_Se_3_ enabled efficient photothermal conversion, making bismuth-based nanoparticles especially promising for combined PTT–RT therapies. Gedda, G. et al. designed gadolinium-doped carbon dots that enabled simultaneous MRI-guided PTT and radiosensitization, demonstrating a synergistic enhancement ratio of 1.53 in a mouse model of colorectal cancer [81]. The integration of imaging capabilities with therapeutic functions in a single nanoplatform represented an important step towards personalized and image-guided cancer therapy, and pre-clinical studies demonstrated the enhanced efficacy of PTT–RT combinations. Eleanor J. Cheadle et al. reported a 71% reduction in tumor volume with gold nanorod-mediated PTT combined with RT in a breast cancer model, compared to 38% and 27% for RT and PTT alone, respectively [82]. Wang et al. found that 67% of the mice treated with the combination therapy of Bi_2_Se_3_ quantum dot–mediated PTT and RT achieved complete tumor eradication [83].

The clinical translation of PTT–RT combinations is in the early stages, with ongoing trials evaluating the combination of gold nanoparticle-mediated PTT with stereotactic body RT in patients with prostate cancer [68]. Preliminary results have shown promising safety profiles and potential efficacy, with several patients exhibiting significant reductions in prostate-specific antigen levels.

### 3.4. Conbination of PTT with Photodynamic Therapy or Sonodynamic Therapy

Photoactivation therapy, which combines PDT and PTT, has emerged as an effective approach for tumor ablation across various cancer types. In this process, photosensitizers and PTAs are critical for successful treatment. PDT enhances tumor cell sensitivity to PTT by modulating the TME, while the heat generated by PTT can stimulate blood flow, improve oxygen delivery, and amplify PDT’s therapeutic effects [84]. While PTT relies on heat to destroy tumor cells, PDT uses light-activated photosensitizers to generate ROS, causing oxidative stress and triggering cell death [85].

**Figure 7:**
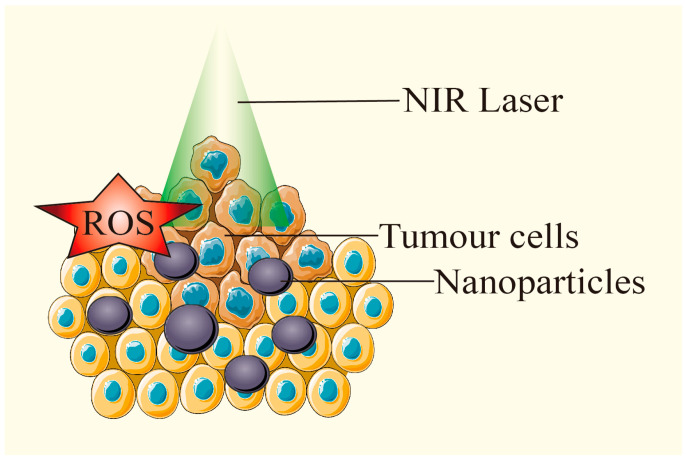
Under laser irradiation, PTAs generate ROS while heating up, and exert PTT and PDT effects at the same time. Adapted from [13], Multidisciplinary Digital Publishing Institute, 2018.

Recent advances in nanotechnology have facilitated the development of nanoplatforms that can act as both photothermal and photodynamic agents. A study developed PTAs as an all-organic nano-drug composed of Protoporphyrin IX (PPIX) and PTAs for the combined treatment of PDT and PTT. The solubility and biocompatibility of PPIX were enhanced by combining it with glycol chitosan. The resulting nanoparticles (PcBu_4_ NPs) demonstrated enhanced cytotoxicity and were able to induce apoptosis in hypoxic A549 cancer cells, showing better efficacy than PTT-only nanoparticles [86]. Lovell et al. developed “porphysome” nanovesicles formed from porphyrin-lipid conjugates, which exhibited excellent photothermal and photodynamic properties [87]. These nanostructures demonstrated high tumor accumulation and effective tumor ablation in vivo. A schematic illustration showed the physiological and biological effects of gold nanoparticle-based PTT and PDT (Figure 7). In this study, the leaky vasculature in tumors led to a high concentration of gold NPs, which, upon exposure to NIR light, induced a photothermal effect. Moreover, a secondary photosensitizer generated ROS, promoting apoptosis and necrosis in the tumor tissue [13]. Li et al. successfully synthesized ICG&Cur@MoS_2_ nanoparticles containing ICG, curcumin (Cur), and molybdenum disulfide (MoS_2_). Their results showed that tumors in the ICG@MoS_2_ + NIR group were significantly smaller than those in the MoS_2_ + NIR group, with lower cell viability in the ICG&Cur@MoS_2_ + NIR group compared to ICG@MoS_2_ + NIR. These NPs not only facilitated PTT and PDT but also inhibited P-glycoprotein, enhancing the PDT effect, making them promising for effective liver cancer treatment [88]. Another group utilized poly (acrylamide-co-acrylonitrile) [P(AAM-co-AN)], gold nanorods (AuNRs), cerium dioxide (CeO_2_), chlorin e6 (Ce6), and pheophorbide A (PA) to prepare nanoparticles P(AAM-co-AN)-AuNRs@CeO_2_-Ce6 (PA/Ce6). AuNRs enabled the nanoparticles to possess high-efficiency photothermal conversion for PTT, with temperature changes regulating Ce6 release. Upon 660 nm laser irradiation, Ce6 generated singlet oxygen to facilitate PDT. After 600 nm and 880 nm NIR irradiation in vitro, both cell viability and migration were reduced. Animal studies showed that PA/Ce6 effectively inhibited tumor growth without causing acute physiological toxicity, suggesting its promising potential in treating hepatocellular carcinoma [89]. Wang et al. [90] developed a composite nano-system with deep penetration and pH responsiveness, enhancing the effectiveness of photothermal and photodynamic therapies (PTT/PDT) for hypoxic tumors. The designed nano-system ([PHC]PP@ HA NPs) constructed small PHC (Porphyrin-derivative Hybrid Complex) NPs embedded in polymer micelles (polyethylene glycol-polyethyleneimine) which were capped with functionalized hyaluronic acid (HA) on dopamine by co-loading Hb and Ce6. The [PHC]PP@ HA nanoparticles were engineered with pH-responsive characteristics, enabling them to maintain a size of about 140 nm in the bloodstream and rapidly release smaller PHC NPs (around 10 nm) for improved tumor penetration within the TME. In vitro studies demonstrated that PHC NPs could penetrate over 110 microns in multicellular tumor spheroids. In vivo anti-tumor results showed that the tumor inhibition rate of [PHC]PP@ HA NPs, which provided Hb for oxygen on demand, reached nearly 100%, significantly outperforming PTT alone or Hb-free nanoparticles ([PC]PP@ HA NPs). After 60 days, the feedback-guided tumor therapy, utilizing PTT/PDT via PHC-based nanoparticles, led to effective tumor ablation and a low recurrence rate of just 8.3%, highlighting the multifunctionality of the PTT/PDT approach. Furthermore, combining PDT and PTT with immune checkpoint inhibitors (e.g., PD-1/PD-L1) can further boost systemic anti-tumor immunity [91].

SDT is developed from PDT, but its tissue penetration is stronger. The mechanism of action is that active oxygen generated by low-frequency ultrasound exciting acoustic sensitizers gathered in tumors kills tumor cells. The high oxidation resistance of hepatocellular carcinoma weakens the efficiency of SDT, and tumor cells may change the sensitivity of SDT under high fever. Li et al. [92] developed a pH-sensitive, self-assembled glycopican-3 binding peptide (GBP) dye based on croconaine dye (CR), namely CR-PEG-GBP, which could be used for PTT/SDT synergistic photoacoustic imaging of liver cancer under the guidance of second near-infrared (NIR-II, 1000~1700 nm) imaging. After CR-PEG-GBP molecules were actively targeted to hepatocellular carcinoma, due to the hydrophilic–hydrophobic transformation induced by the acidic tumor microenvironment, the molecules self-assembled into large-sized nanoparticles in situ, which greatly improved the retention rate of particles in the tumor and enhanced the photoacoustic ablation efficiency in the treatment of hepatocellular carcinoma. This study provided a simple and effective solution for the treatment of liver cancer, and the synthesis process of CR-PEG-GBP probe was simple and had great clinical application potential.

### 3.5. Combination of PTT with Gene Therapy

Gene therapy involves changing the biological characteristics of living cells by modifying or manipulating gene expression to achieve therapeutic objectives. However, nucleic acids are easily degraded by biological enzymes, and they face many obstacles when entering the target cells. Gene vectors can protect therapeutic nucleic acids from being degraded by enzymes and transport them into the target cells [93]. The modified PTAs can be used as gene carriers, which can be internalized by cells and generate heat under NIR irradiation, so that the endosome can be rapidly ruptured, which can not only kill tumor cells but also improve the gene transfection effect.

**Figure 8:**
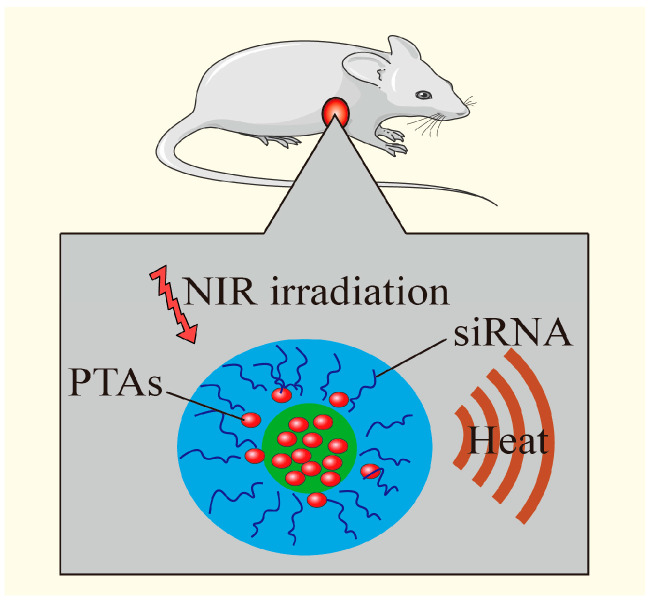
PTT increases the temperature, kills tumor cells, destroys the chemical bond between genes and nanomaterials, promotes gene release, and achieves the combined effect. Adapted with permission from [14], American Chemical Society, 2011.

In recent years, the rapid development of nano-materials has promoted the rapid progress of gene therapy. The photothermal effect can kill tumor cells and promote gene release by breaking the chemical bond connecting genes and nano-materials. Moreover, the localized heating induced by PTT can trigger the expression of heat-responsive genes, providing temporal and spatial control for gene therapy (Figure 8) [14]. Yan et al. successfully constructed a novel AuNCs/PEI/miRNA/HA complex by using gold nanoclusters (Au NCs), polyethylenimine (PEI), microRNA (miRNA), and HA through layer-by-layer self-assembly technology, achieving the target of miRNA through HA receptor-mediated endocytosis. They observed that this complex not only effectively improved the efficiency of gene transfer but also regulated gene expression when miRNA was interfered with. The laser irradiation compound can produce a better therapeutic effect on hepatocellular carcinoma compared with single therapy [94]. Liu et al. developed a multifunctional nanostructure, GAL-GNR-siGPC-3, by integrating galactose (GAL), gold nanorods (GNR), and siGPC-3 genes. In vitro studies demonstrated that this nanostructure effectively delivered siRNA to hepatocellular carcinoma cells, silenced the GPC-3 gene, and suppressed tumor cell growth. Additionally, GAL-GNR exhibited significant tumor-killing ability when exposed to NIR irradiation, while GAL-GNR-siGPC-3 showed a synergistic effect in liver cancer treatment [95]. Meanwhile, Yin et al. utilized GO nanosheets modified with FA, NH_2_-mPEG-NH_2_ (5 k), and poly-allylamine hydrochloride as a platform for combined photothermal and gene therapy in pancreatic cancer. This system carried KrasI (a K-ras gene inhibitor) and was encapsulated with a lipid bilayer to improve stability, biocompatibility, and photothermal efficiency. The use of polymers to modify GO enhanced its water solubility and its ability to efficiently deliver siRNA. At the same time, the use of FA molecules to modify GO greatly improved the tumor site targeting of the GO complex. The study showed that the modified GO could simultaneously deliver siRNA of the HDAC1 and KRas1 genes to the pancreatic cancer cell MIA PaCa-2 with high selectivity, as well as inhibit the expression of HDAC1 and Kras genes. The tumor inhibition rate in vivo reached more than 80% without any toxic or side effects [96].

### 3.6. Combination of Chemodynamic Therapy with PTT

CDT can trigger the Fenton or Fenton-like reaction of tumor cells in a weakly acidic TME and generate hydroxyl radicals to kill tumor cells by using metal nanoparticles to catalyze endogenous hydrogen peroxide. However, CDT is mainly limited by the low efficiency of the Fenton reaction when it is used in vivo, and PTT can raise the temperature of TME by raising the local temperature. The synergistic treatment of CDT and PTT can not only compensate for the limitation of PTT efficiency caused by insufficient light penetration but also improve the efficiency of the Fenton reaction [97].

Dang et al. created a 3D-printed hydrogel scaffold, gel-SA-CuO, incorporating hydrogel (gel), sodium alginate (SA), and copper oxide (CuO). CuO nanoparticles serve as a reservoir, releasing Cu^2+^ in acidic environments, which triggers intracellular ROS generation via a Fenton-like reaction. Additionally, CuO acts as a PTA, producing heat to enhance the efficiency of the Fenton-like reaction. Furthermore, the released Cu^2+^ leads to glutathione depletion and glutathione peroxidase 4 inactivation, thereby inducing ferroptosis to kill cancer cells. Implanting the hydrogel stent at the resection site inhibited the postoperative recurrence of liver cancer, which provided a new insight for eliminating the postoperative recurrence of cancer [98]. Yang et al. synthesized V-ION-VIO by using new vanadium (V), iron oxide [Iron(III) oxide, ION] and nanoparticles (VIO), which were loaded with sorafenib (SF) to form VIO/SF and coupled with transferrin (TF) to form a TVIO/SFD nano-preparation. The VIO-based nano-preparation had a significant inhibitory effect on liver cancer cells, was able to induce a Fenton-like reaction to induce cell apoptosis and ferroptosis, and also exhibited high photothermal conversion efficiency. NIR-activated TVIO had a significant tumor clearance effect in a mouse liver cancer tumor model. In the study, VIO not only showed anti-angiogenesis potential but was also able to be used as an excellent MRI probe to obtain high-resolution diagnostic images. This VIO nano-platform combined with CDT, photothermal, and diagnostic capabilities is a potential candidate drug for the treatment and diagnosis of liver cancer [99]. Xu et al. wisely designed the glucose-responsive enzyme Fe@HRP-ABTS/GOx nanodots for tumor-specific PTT/CDT. They demonstrated that glucose oxidase (GOx) oxidized glucose in tumor cells, increasing H_2_O_2_ levels at the tumor site, which activated a glucose-based tumor therapy. The self-produced H_2_O_2_ then converted ABTS into oxidized ABTS (oxABTS) via horseradish peroxidase. Additionally, Fe^2+^ generated via the GSH-mediated reduction of Fe^3+^ reacted with H_2_O_2_ to produce highly reactive OH radicals through the Fenton reaction, depleting GSH and promoting effective CDT. Both in vitro and in vivo tests showed that Fe@HRP-ABTS/GOx nanoparticles effectively killed cancer cells and eradicated tumors under NIR laser treatment [100]. Wen et al. developed Cu_2_O nanoparticles with a quasi-circular shape, approximately 100 nm in diameter, that increased in temperature from 25 °C to 50 °C upon NIR light (0.5 W/cm^2^) irradiation for 5 min. At a near-neutral pH of 6.5, these nanoparticles significantly generated ROS, inhibiting gastric cancer cell proliferation, invasion, and migration in a concentration-dependent manner [101]. In Zhang’s study, hollow mesoporous silica nanoparticles (HMSNs) were used to deliver Cu(II)-doped polydopamine (PDA), forming the HMSNs@PDA-Cu platform for synergistic therapy. PDA acted as a photothermal agent (PTA) for PTT, while Cu(II) interacted with intracellular glutathione (GSH) to produce Cu(I), which then reacted with H_2_O_2_ to generate toxic hydroxyl radicals (•OH) through a Fenton-like process. The Cu(II)–PDA combination improved photothermal efficiency, while PDA exhibited superoxide dismutase (SOD)-like activity, converting O^2–^ to H_2_O_2_ and enhancing CDT. The elevated temperature from PTT further boosted •OH production, enhancing CDT. This nanotherapeutic platform showed effective tumor reduction in mice, highlighting strong potential for cancer metastasis treatment both in vitro and in vivo [102].

### 3.7. Combination of PTT with Other Therapies

#### 3.7.1. PTT Combined with Gas Therapy

Gas therapy has gained increasing attention as a promising strategy in cancer treatment. Various therapeutic gases, including carbon monoxide, nitric oxide, hydrogen sulfide, oxygen, and sulfur dioxide, play key roles in regulating cellular, tissue, and organismal processes, showing significant potential in cancer therapy. Gas therapy can address the limitations of PTT in terms of treatment efficiency, enabling a synergistic effect where “1 + 1 > 2” Ma et al. developed a novel amphiphilic gas carrier material, mPEG (CO), derived from carbonyl iron, and created an aggregation induced emission (AIE) nano-drug delivery system. This system, triggered by the TME, was formed via co-assembling with an AIE polymer photothermal material that emits NIR light. When nano-bombs met with over-expressed H_2_O_2_ in the TME, carbon monoxide gas was released quickly. Surprisingly, this released carbon monoxide inhibited the rapid proliferation of tumor cells and reduced the over-expression of heat shock proteins during PTT with AIE materials, thereby enhancing the effectiveness of PTT at lower temperatures [103].

#### 3.7.2. PTT Combined with Hunger Therapy

Hunger therapy can achieve the goal of “starving” tumor cells by blocking the nutritional supply of tumors. Combined with PTT, tumor cells can be further killed by thermal effect to achieve synergistic therapeutic effect. GOx efficiently catalyzes the oxidation of glucose to produce gluconic acid and H_2_O_2_. By selectively depleting glucose in tumors, GOx can effectively starve the tumor of its energy supply, offering a potential strategy for hunger-based therapy. Combining GOx-mediated starvation with other therapeutic approaches and exploring multi-modal treatments could provide novel strategies for cancer therapy. Fu et al. loaded the hollow mesoporous silicone nanoparticles with GOx and arginine and carried out in vivo tumor hunger treatment based on GOx for the first time, providing a new concept for the research of hunger therapy based on a GOx catalytic reaction combined with PTT in cancer multi-mode synergistic therapy [104]. The non-specific “always-on” nature of traditional NIR-II PTA and tumor metastasis related to PTT limits the progress of PTT. In the designed cascade bioreactor (LGT), based on clinically applicable liposome nanoparticles, the protophotothermal agent is converted in situ within the TME into a NIR-II absorption charge transfer complex (CTC) through glucose consumption and H_2_O_2_ production catalyzed by GOx. This glucose deprivation not only reduces the tumor’s energy supply but also increases the sensitivity of LGT to photothermal ablation. When exposed to NIR-II laser irradiation, LGT-mediated in-situ PTT effectively ablates the primary tumor and further exposes tumor-associated antigens [105].

### 3.8. Multi-Modal Therapy Based on PTT

Apart from the above-mentioned double synergistic therapy, photothermal three-mode synergistic therapy has also been part of the latest research progress, which can enhance the anti-tumor effect with more complementary therapies.

#### 3.8.1. PTT Combined with PDT and Chemotherapy

Wang et al. developed FA-GT-MSNs@TPZ nanoparticles with a Janus structure using FA, gold nanotriangle (GT), mesoporous silica nanoparticles (MSNs), and tirapazamine (TPZ). The FA receptor-targeting ligand boosted the internalization of the Janus nanoplatform in hepatocellular carcinoma cells, which exhibited radiation sensitivity and photothermal toxicity. The pH-sensitive release of its loaded hypoxia-activated prodrug, Tirazamine, further enhanced the anti-tumor effect in a synergistic manner. Experiments showed that the Janus nanoplatform effectively combines radiosensitization, local PTT, and hypoxia-targeted chemotherapy, achieving promising results in both in vitro and in vivo models with minimal side effects. This approach demonstrates the potential for synergistic liver cancer treatment through hypoxia-activated RT, chemotherapy, and PTT [106]. Yu et al. designed and synthesized zinc phthalocyanine (ZnPc) and sorafenib (SFB) nanoparticles coated with bovine serum albumin (BSA). ZnPc/SFB@BSA exhibited strong NIR absorption, enabling its use in PTT and PDT, while SFB significantly inhibited hepatocellular carcinoma metastasis. The results showed that the ratio of ZnPc to SFB was 4:1, which may improve the curative effect of triple therapy and reduce the adverse reactions of SFB. After 730 nm laser irradiation, ZnPc/SFB@BSA could significantly inhibit the proliferation and metastasis of hepatocellular carcinoma cells in vitro and promote apoptosis, while the intravenous injection of ZnPc/SFB@BSA could significantly reduce the tumor growth in nude mice orthotopic xenotransplantation liver cancer model. The nanoparticles also had low toxicity and good blood compatibility, providing a synergistic PDT/PTT/chemotherapy system for the treatment of liver cancer [107].

#### 3.8.2. PTT Combined with PDT and RT

Liu et al. used aspartic acid (RGD), polyethylene glycol (PEG), polyacrylic acid (PAA), metronidazole (MN)—which could enhance the radiosensitivity to hypoxic tumor tissues—and liquid metal (LM) to prepare RGD-PEG-PAA-MN@LM. Under near infrared and X-ray continuous radiation, composite nanoparticles produced more ROS, which killed tumor cells more significantly. In in vivo experiments with tumor-bearing mice, the tumor inhibition effect of RGD-PEG-PAA-MN@LM after two near-infrared and X-ray treatments for 14 days was obviously better than the liquid metal particle group, which showed that the composite nanoparticles had a remarkable synergistic effect and could provide a unique method for the combined treatment of PTT, PDT, and RT in liver cancer [108].

#### 3.8.3. PTT Combined with CDT and Chemotherapy

Wu et al. designed a combined therapy system (ICPs@PDA:CuO_2_NPS) for the PC-3 tumor model, integrating a high-efficiency photothermal agent, poly-dopamine (PDA-Fe), the chemokinetic agent CuO_2_, and a synergistic chemotherapy agent, the adriamycin-gossypol infinite coordination polymer (ICP). The size of the composite nanoparticles was 116.45 ± 18.32 nm, and the photo-thermal conversion efficiency was as high as 52.4%. The composite nanoparticles could specifically generate free radicals in tumor cells for chemodynamic treatment and eliminate glutathione. The loaded ICPs released two chemotherapeutic drugs with different mechanisms in tumor cells. The system made PTT, CDT, and chemotherapy activate at different times, resulting in an excellent synergistic effect and achieving nearly 100% tumor inhibition with the lowest power and minimum drug dosage. The tumors of the mice did not recur within 60 days after treatment [49]. All in all, the photothermal/chemodynamics/chemotherapy synergistic therapy has the characteristic of a programmable design. Once initiated, it can be automatically executed, allowing the proportion of different therapeutic agents and treatment schemes in the system to be adjusted accordingly as needed. Different programmed three-mode treatment strategies are adopted for different tumors to achieve accurate and personalized treatment.

#### 3.8.4. PTT Combined with Gene Therapy and Immunotherapy

Lu et al. designed a multifunctional layered double hydroxide nanoplatform based on pH-sensitive RNA-small interfering RNA. After laser irradiation, the thermal effect of layered double hydroxide changed the “cold” TME and enhanced the synergistic immunotherapy of NR2F6-siRNA and anti-PD-L1. The results showed that this multifunctional nano-platform could significantly inhibit the growth of irradiated primary tumor and non-irradiated distant tumor. In addition, the immune response stimulated by local treatment could also make the whole body establish long-term immune memory, thus inhibiting tumor metastasis. This was considered as a proof of concept for targeting surface and intracellular immune checkpoints and complemented the existing immunotherapy for liver cancer [109].

The combination of PTT with other treatment modalities can enhance anti-tumor effects, but there are still some limitations: (1) The penetration depth of light is limited, especially in the treatment of deep tumors; (2) Damage to normal tissues still occurs. Although nanotechnology can improve targeting, tumor heterogeneity makes treatment effects unstable; (3) Carriers still need further optimization, as drug delivery systems may face issues such as unstable release rates or carrier toxicity; (4) Accurate control of PTA dosage is a challenge. Excessive heating can lead to tissue damage, while insufficient dosage may not achieve therapeutic effects; (5) The complexity of combination therapies increases treatment costs and may also add side effects; (6) Although PTT helps to regulate the TME, tumor immune escape mechanisms still pose a challenge for combined immunotherapy. In summary, the combined use of PTT with other treatments still requires further optimization in terms of targeting, penetration depth, drug resistance, treatment duration, and safety.

## 4. Conclusions and Perspectives

PTT harnesses PTAs to transform light energy into thermal energy. This process not only elicits local hyperthermia for the ablation of tumor cells but also augments drug delivery efficiency by capitalizing on the elevated temperature. Increased temperatures accelerate the release of drugs from drug delivery systems. When combined with chemotherapy or RT, PTT can improve the tumor’s absorption of the chemotherapeutic agents, enhance the cytotoxicity of these drugs, and increase the radiosensitivity of tumor cells, thus improving the effectiveness of both chemotherapy and RT. Additionally, when combined with CDT, PTAs are activated by laser at the tumor site, promoting the production of free radicals and boosting the therapeutic impact of CDT. Moreover, the thermal effect can also accelerate the release of gases associated with gas therapy, and the combination of the two therapies improves tumor blood flow, inhibits tumor growth, and promotes immune responses, while also suppressing tumor growth and stimulating immune responses. Additionally, the ROS generated subsequent to PTA activation can further induce tumor cell apoptosis. When integrated with gene therapy, PTT promotes the absorption of genetic drugs through its thermal effect. Meanwhile, the thermal effect improves the absorption of gene drugs, which enhance the sensitivity of tumor cells to PTT by regulating specific genes, remarkably improving the overall anti-tumor efficacy. In general, PTT in combination with other therapeutic modalities (such as chemotherapy, RT, gene therapy, PDT, gas therapy, CDT, multimodal therapy, photoacoustic imaging, and starvation therapy) exhibits high efficiency, synergistic effects, and favorable biocompatibility, rendering it a promising, novel approach for eradicating tumor cells with substantial application potential.

The clinical implementation of PTT combined with other tumor treatments faces several challenges, including operational difficulties, regulatory barriers, scalability issues in nanoparticle production, and concerns about cost-effectiveness. There are several measures that can be taken to address these challenges. For example, photoacoustic imaging has been used to monitor the real-time effects of PTT treatment, which helps minimize the risk of damage to healthy tissues. Additionally, early communication with regulatory agencies, such as the FDA, during the research and development phase ensures that the treatment methods meet regulatory standards, which helps avoid delays in the approval process and shortens approval times. Furthermore, nanoparticle production requires precise control, which is often insufficient for large-scale clinical application. Advances in microfluidic technology have improved the consistency and controllability of nanoparticle production, helping to solve this issue. The cost of nanoparticles is relatively high, but using low-cost materials, such as carbon nanotubes instead of gold nanoparticles, helps reduce costs. As for the potential toxicity of nanoparticles in vivo, the development of biocompatible and biodegradable nanocarriers to reduce toxicity has become a key research direction. In addition, the development of low-toxicity, biodegradable photothermal agents with targeting capabilities also represents a promising direction. Artificial intelligence is also playing an increasingly important role in PTT combination therapy, driving the precision and personalization of oncology treatment by supporting disease diagnosis, treatment planning, and efficacy evaluation. AI can help doctors evaluate treatment effects, optimize treatment regimens, predict disease risk and adjust treatment in advance, especially in PTT combination therapy. It can also simulate drug molecules and screen the most likely treatment regimens, thereby accelerating drug development, and is expected to help discover new photothermal agents or combination drugs to improve tumor treatment.

In summary, although the clinical implementation of PTT combined therapy faces numerous challenges, the aforementioned cases demonstrate that with the relentless effort of researchers in related fields, technological advancements, and multi-party collaboration, these issues may be effectively addressed. This also indicates that PTT combined therapy based on nanoplatforms represents the forefront of cancer treatment, capable of overcoming the limitations of traditional therapies, providing higher efficacy and fewer side effects.

## Figures and Tables

**Table 1:** Classification, absorption wavelength range and characteristics of PTAs.

Categories (Main Absorption Wavelength)	Common Example	Characteristics
Metallic nanomaterials (500–1200 nm)	Gold NPs, Silver NPs	Excellent photothermal conversion efficiency. Conversion mechanism primarily relies on surface plasmon resonance Biocompatibility enhanced through surface modification. Can be multifunctionalized, such as coupling with targeting molecules for targeted PTT or loading with therapeutic agents for combination treatments [20,21].
Carbon-based nanomaterials (600–2000 nm)	Carbon Nanotubes, Carbon Quantum Dots	High photothermal conversion efficiency allows rapid light-to-heat conversion. Good chemical stability ensures structural integrity in vitro and in vivo. Can be functionalized by attaching bioactive molecules through covalent or non-covalent bonds for combined photothermal-drug or targeted therapy [22,23].
Organic Dyes (650–1000 nm)	IR780 dye, ICG	Good light–absorbing capabilities, and converting it into heat. Certain biocompatibility in vivo. Some organic dyes, in addition to photothermal properties, also have fluorescence characteristics, which can be used for imaging–guided PTT. Stability can be improved by appropriate chemical modifications [24].
Conjugated Polymers (400–1100 nm)	Polystyrene, Poly (3, 4-vinyldioxylthiene -phenylethylene)	Excellent photothermal conversion performance. Structure can be designed and optimized through chemical synthesis to enhance photothermal performance, biocompatibility, and targeting. Can be combined with NPs and biomolecules to create multifunctional agents for combined treatments [25,26].

**Table 2:** Common nanoplatforms and their respective classifications and characteristics.

Common Nanoplatform	Classification	Characteristics	Form and Size
Polymeric Nanoparticles	Biodegradable polymeric NPs and non-biodegradable polymeric NPs	High Customization: Precise control over size, shape, surface properties, and drug release. Good Stability: Protects drugs from degradation and inactivation. Sustained and Controlled Release: Enables slow or specific release patterns (e.g., pH or enzyme-sensitive). Surface Modification: Easy conjugation of targeting molecules for multifunctionality [36].	Generally spherical or nearly spherical, in the range of tens to hundreds of nanometers.
Liposomal Nanoparticles	Traditional liposomes, long-circulating liposomes, targeted liposomes, etc.	Good Biocompatibility: Compatible with biological cells, reducing immunogenicity and toxicity. Drug Loading Capacity: Enhances drug solubility. Targeting: Surface modification enables active targeting, increasing drug accumulation at tumor sites. Sustained Release: Delays drug release, prolongs circulation and action time, reducing administration frequency [37].	Usually spherical in shape, in the range of 30 to 1000 nm.
Micellar Nanoparticles	Block copolymer micelles, graft copolymer micelles, etc.	Unique Core-Shell Structure: Improves the solubility and bioavailability of drugs. Small Particle Size: Usually between 10–1000 nm, which is beneficial for penetration and diffusion in vivo, and is easier to enter the tumor tissue. Good Physical Stability: Has good stability. Targeted Delivery: Achieves specific targeting of tumor cells [38].	Usually spherical in shape, in the range of 10 to 100 nm.
Inorganic Nanoparticles	Gold NPs, graphene, quantum dots, silver NPs, carbon nanotubes, etc.	Unique Physical Properties: Gold NPs offer good optical properties for PTT and imaging; quantum dots excel in fluorescence for bioimaging and diagnosis. Large Specific Surface Area: Can carry large amounts of medicine and allow extensive surface modification. Multifunctional Design: Integrates drug loading, targeting, and imaging functions. Potential Toxicity: Some inorganic NPs may exhibit toxicity [20].	Usually spherical or polyhedral, ranging from 1 to 100 nm.
Virus-Like Nanoparticles	Nanomedicine modified by natural virus, synthetic virus-like NPs, etc.	Natural Origin: Good biocompatibility, low immunogenicity, and high safety. Transmission of Biological Information: Carries bioactive substances, regulates TME and immune response. Targeting Ability: Surface modification or natural targeting enables specific delivery to tumor cells. Stability: Relatively poor stability, requiring stringent storage conditions [39].	Similar to the geometry of a virus, usually spherical or polyhedral, in the range of 20 to 200 nm.
Extracellular Vesicle Nanoparticles	Exosomes, microvesicles, etc.	Natural Origin: Low toxicity and good biocompatibility. Transmission of Biological Information: Carries bioactive substances, participates in immune responses, and regulates the TME. Targeting Ability: Natural targeting properties can be enhanced by modification. Stability: Relatively poor, and requires special preservation methods [40].	Usually spherical or elliptical, in the range of 30 to 1000 nm.

**Table 3:** The mechanisms and characteristics of PTT combined with different therapeutic methods.

Common Combination Mode	Classification	Mechanism and Characteristics
Combination of PTT with Chemotherapy	Promote drug uptake and accumulation	PTT can enhance tumor vascular permeability, improve drug uptake, and promote the accumulation and release of drugs at the tumor site, thus improving the efficacy of anti-tumor therapy.
	Synergy of anti-tumor effects	PTT combined with chemotherapy can improve tumor targeting, promote drug release, and produce a synergistic therapeutic effect.
	Enhancement of the responsiveness of tumor cells to chemotherapy drugs	PTT changes the toxicity of chemotherapy drugs and enhances the effectiveness of chemotherapy by regulating temperature. PTT can also enhance the sensitivity of cancer cells to drugs by regulating DNA repair mechanisms.
	Overcome of multidrug resistance	By destroying mitochondrial function, PTT inhibits the production of ATP, reduces drug efflux, and improves the effectiveness of chemotherapy drugs against drug-resistant cancer cells.
Combination of PTT with Immunotherapy	PTT in combination with immune adjuvants	The therapy has the potential to enhance anti-tumor immunity, induce apoptosis of tumor cells, alleviate immunosuppression, and prevent recurrence and metastasis.
	PTT in combination with immune checkpoint inhibitors	The therapy enhances tumor targeting, immune response, and anti-tumor efficacy, and has shown promising results in preclinical studies and early clinical trials of advanced solid tumors.
Combination of PTT with RT	Increase of radiosensitivity	PTT-induced hyperthermia can improve the sensitivity of tumor cells to radiation by inhibiting DNA repair mechanisms and increasing oxidative stress.
	Overcoming of radioresistance	PTT leads to protein denaturation and membrane destruction, while RT induces DNA damage. The combination of the two can help overcome resistance mechanisms and improve the overall therapeutic effect.
	Development of some advanced nanoplatforms	This advanced nanoplatform has shown significant synergistic enhancement in PTT combined with RT sensitization, enhancing the efficacy of cancer treatment, and preclinical studies have demonstrated its potential safety and efficacy.
Conbination of PTT with PDT or Sonodynamic Therapy(SDT)	Combination of PTT with PDT	PDT enhances the sensitivity of tumor cells to PTT by modulating the TME, while the heat generated by PTT can stimulate blood flow, improve oxygen delivery, and amplify the therapeutic effects of PDT. This combined effect promotes tumor cell death through both heat and ROS.
	Conbination of PTT with SDT	Low frequency ultrasound stimulates the acoustic sensitizers gathered in the tumor to produce reactive oxygen species to kill tumor cells.
Combination of PTT with Gene Therapy		PTT can break the modified gene vector, promote the regulation of gene release and expression, and exert the therapeutic effect of PTT.
Combination of Chemodynamic Therapy (CDT) with PTT		PTT improves the efficiency of the Fenton reaction by increasing the temperature of the TME, and collaborates with CDT for anti-tumor.
Combination of PTT with Other Therapies	PTT combined with gas therapy	Gas therapy can enhance the efficacy of PTT at low temperatures, inhibit the proliferation of tumor cells, and achieve synergistic effects.
	PTT combined with hunger therapy	Starvation therapy limits the glucose supply to tumors, and the combination with PTT can not only “starve” tumor cells but also enhance the therapeutic effect through the thermal effect.
Multi-Modal Therapy Based on PTT	PTT combined with PDT and chemotherapy	The synergies of PTT and other therapies work together through multiple mechanisms to enhance treatment effectiveness, reduce drug resistance, and minimize side effects
	PTT combined with PDT and RT	
	PTT combined with CDT and chemotherapy	
	PTT combined with gene therapy and Immunotherapy	

## List of Abbreviations

AIE	aggregation induced emission
AP3	ansamitocin P3
ATP	adenosine triphosphate
BP	black phosphorus
BSA	bovine serum albumin
CDT	chemodynamic therapy
Ce6	chlorin e6
CeO_2_	cerium dioxide
CuO	copper oxide
Cur	curcumin
DAMPs	damage-associated molecular patterns
DOX	doxorubicin
Er	erlotinib
FA	folic acid
GAL	galactose
GBP	glycopican 3 binding peptide
gel	incorporating hydrogel
GO	graphene oxide
GOx	glucose oxidase
GSH	glutathione
HA	hyaluronic acid
Hb	hemoglobin
HMSNs	hollow mesoporous silica nanoparticles
ICG	indocyanine green
ICPs	infinite coordination polymer
LM	liquid metal
MGO	modified graphene oxide
MN	metronidazole
mPDA	mesoporous polydopamine
MPE	maximum permissible exposure
MSNs	mesoporous silica nanoparticles
NIR	near-infrared
NPs	nanoparticles
PA	pheophorbide A
PAA	polyacrylic acid
PBA	phenylboronic acid
PBNPs	prussian blue nanoparticles
PDT	photodynamic therapy
PEG	polyethylene glycol
PEI	polyethylenimine
PHC	porphyrin-derivative hybrid complex
PPIX	protoporphyrin IX
PTAs	photothermal agents
PTT	photothermal therapy
RBL1/p107	retinoblastoma-like protein 1
RGD	aspartic acid
ROS	reactive oxygen species
RT	radiotherapy
SA	sodium alginate
SDT	sonodynamic therapy
SFB	sorafenib
SOD	superoxide dismutase
TCA	thyrocalcitonin
TF	transferrin
TME	tumor microenvironment
TPZ	tirapazamine
V	vanadium
VIO	nanoparticles
ZnPc	zinc phthalocyanine
